# Additive Effects of Cognitive Remediation and Physical Exercise in Patients with Schizophrenia: Cognitive Improvements

**DOI:** 10.3390/brainsci16090933

**Published:** 2026-08-31

**Authors:** Diego Berrezueta, Juan P. Espinoza, Ricardo Arroyo, Benjamín Cartes, Karina Venegas, Rodrigo R. Nieto

**Affiliations:** 1 Escuela de Medicina, Facultad de Medicina, Universidad de Chile, Santiago 8380453, Chile; 2 Clínica Psiquiátrica Universitaria, Hospital Clínico de la Universidad de Chile, Universidad de Chile, Santiago 8380453, Chile; 3 Departamento de Psiquiatría y Salud Mental Norte, Facultad de Medicina, Universidad de Chile, Santiago 8380453, Chile; 4 Departamento de Neurociencias, Facultad de Medicina, Universidad de Chile, Santiago 8380453, Chile

**Keywords:** schizophrenia spectrum disorders (SSDs), psychosis, neurocognition, cognitive deficit, cognitive impairment, cognitive training, cognitive enhancement, psychiatric rehabilitation, physical activity

## Abstract

**Highlights:**

**What are the main findings?**

- Combining cognitive remediation and physical exercise seems to provide better cognitive results than each intervention by itself.
- This observation was found for global cognitive composite scores and some cognitive domains, such as verbal memory and executive function.

**What are the implications of the main findings?**

- Future guidelines could recommend combining cognitive remediation and physical exercise for better cognitive outcomes, as this is not yet the current standard clinical practice.
- Potential shared molecular mechanisms should be studied, as both cognitive remediation and physical exercise can increase the expression of brain-derived neurotrophic factor (BDNF).

**Abstract:**

Cognitive impairment is a clinically important feature of schizophrenia that affects community functioning and responds poorly to pharmacological treatment. Cognitive remediation (CR) and physical exercise (PE) can each improve cognition, but whether their combination provides additive benefits remains uncertain. This systematic review examined cognitive outcomes of interventions combining CR and PE in adults with schizophrenia or other psychotic spectrum disorders. The protocol was registered in PROSPERO (ID: 639266). PubMed was searched for eligible studies, and findings were synthesized narratively because of heterogeneity in designs, interventions, comparators, and outcome measures. Risk of bias was assessed using RoB 2 for randomized trials and ROBINS-I for non-randomized studies. Eleven reports representing 10 underlying studies met the eligibility criteria. Combined CR and PE was associated with improvements in global cognitive performance and in domains including executive function, verbal memory, working memory, and processing speed. Some studies reported benefits over CR alone or PE alone, while three-arm trials provided the most direct, although not uniformly consistent, evidence of superiority over both monotherapies. Four randomized reports raised some concerns regarding risk of bias; three were judged to be at high risk, and all four non-randomized reports were judged to be at serious risk. The single-database search, methodological heterogeneity, and risk-of-bias concerns limit the certainty and generalizability of the findings. Combined CR and PE may provide additional cognitive benefits, but adequately powered trials with appropriate comparators and standardized outcomes are needed to establish additivity.

## 1. Introduction

Schizophrenia is a severe psychiatric disorder characterized by psychotic symptoms, such as delusions and hallucinations; negative symptoms, such as avolition and anhedonia; and cognitive impairments affecting executive function, processing speed, learning, and memory [1,2]. An appropriate treatment plan includes not only antipsychotic medication but also psychological therapies, social support, and rehabilitation strategies. Nevertheless, there remains a pressing need for more effective treatments and improved delivery of services [2,3].

Cognitive deficits in schizophrenia have been demonstrated across different regions of the world, despite linguistic and cultural differences [4,5]. These deficits are particularly relevant because they substantially affect community functioning and remain an important clinical target that has proven difficult to address through pharmacological treatment alone [6,7].

Cognitive remediation (CR) is a non-pharmacological intervention that has demonstrated efficacy in treating cognitive deficits in schizophrenia [8,9]. CR can be delivered individually through computer-based programs or in group formats. It aims to improve cognitive performance through repeated practice targeting specific cognitive domains, known as a restorative approach, or through the use of cognitive strategies and environmental accommodations to compensate for cognitive impairment, known as a compensatory approach [10,11]. The overall effect of cognitive remediation on cognition has been reported to be small to moderate (d = 0.29; 95% CI, 0.24–0.34), although its effects may vary across cognitive domains [9].

Physical exercise (PE) is another non-pharmacological approach for addressing cognitive deficits in people with schizophrenia [8]. It can be considered an evidence-based adjunctive intervention because it provides consistent benefits for people living with mental disorders. In schizophrenia, physical activity is recommended as part of rehabilitation programs to improve symptom severity and overall quality of life [12]. Evidence also supports the efficacy of physical activity, particularly aerobic exercise, in improving cognitive functioning in people with schizophrenia [13].

In recent years, some authors have suggested combining physical exercise and cognitive remediation to obtain greater benefits [12,14,15], based on the improvements that each intervention has individually shown in the cognitive functioning of people with schizophrenia. Shared molecular mechanisms of CR and PE, such as the promotion of brain-derived neurotrophic factor (BDNF) expression [16], provide further neurobiological support for this combined approach.

Deste et al. previously provided a qualitative critical review of physical exercise, both alone and in combination with cognitive remediation, and discussed seven studies examining combined interventions [12]. However, that review did not employ a systematic study-selection process or formally assess the risk of bias of the included studies. The present review extends this earlier work by following a registered protocol, applying explicit eligibility criteria and PRISMA-based study selection, incorporating more recent evidence, including two three-arm randomized trials published in 2025, and evaluating the included reports using design-specific risk-of-bias tools.

For the purposes of this review, superiority of CR + PE over both CR alone and PE alone was regarded as the most direct evidence of an additive effect. Superiority over only one of these interventions was interpreted as an incremental benefit relative to that particular comparator and was not, by itself, considered sufficient to establish additivity. Synergy would require evidence that the effect of combining CR and PE exceeded that expected from their individual effects, generally assessed through a formal interaction analysis.

Using the PICO framework, the review question was whether, among adults with schizophrenia or other psychotic spectrum disorders (population), combined cognitive remediation and physical exercise (intervention), compared with cognitive remediation alone, physical exercise alone, or another active or non-active condition (comparators), produces greater improvements in global or domain-specific cognitive performance (outcomes). We hypothesized that combined CR and PE would outperform CR alone and PE alone on global cognitive composite scores and on specific cognitive domains, particularly executive function and verbal memory.

## 2. Materials and Methods

### 2.1. Protocol and Registration

This systematic review was conducted in accordance with the Preferred Reporting Items for Systematic Reviews and Meta-Analyses (PRISMA 2020) guidelines [17]. The PRISMA checklist has been published as Supplementary Material (Supplementary File S1). The review protocol was registered in the International Prospective Register of Systematic Reviews PROSPERO (registration ID: 639266).

### 2.2. Eligibility Criteria

Eligible studies had to include adults aged 18 years or older diagnosed with schizophrenia, schizoaffective disorder, or another psychotic spectrum disorder. Studies with mixed diagnostic samples were eligible only when data for participants with a psychotic spectrum disorder were reported separately and could be independently extracted. Studies were required to evaluate an intervention combining cognitive remediation and physical exercise and to include either a comparator group—such as standard treatment, no additional intervention, or an alternative therapy—or a pre–post assessment of the intervention group. We excluded studies conducted exclusively in participants without a psychotic spectrum disorder, studies involving pediatric populations or participants younger than 18 years, and studies evaluating pharmacological interventions or non-pharmacological interventions that did not include both cognitive remediation and physical exercise. Additionally, reporting cognitive outcomes was an inclusion criterion.

### 2.3. Information Sources and Search Strategy

A systematic literature search was conducted in PubMed to identify studies evaluating interventions combining cognitive remediation and physical exercise in individuals with schizophrenia or other psychotic spectrum disorders. No language restrictions were applied. The search strategy combined terms related to schizophrenia and psychotic disorders with terms for cognitive remediation and physical exercise. The search string was ((“Schizophrenia”[Title/Abstract]) OR (“Schizophrenia”[MeSH Terms]) OR (“Psychotic Disorders”[Title/Abstract]) OR (“Psychotic Disorders”[MeSH Terms]) OR (“Psychosis”[Title/Abstract])) AND ((“Cognitive Remediation”[MeSH Terms]) OR (“Cognitive Rehabilitation”[Title/Abstract]) OR (“Cognitive Remediation”[Title/Abstract]) OR (“Cognitive Training”[Title/Abstract]) OR (“Cognitive Enhancement”[Title/Abstract])) AND ((“Exercise”[MeSH Terms]) OR (“Exercise”[Title/Abstract]) OR (“Physical Exercise”[Title/Abstract]) OR (“Exercise Therapy”[MeSH Terms]) OR (“Exercise Therapy”[Title/Abstract]) OR (“Resistance Training”[MeSH Terms])).

### 2.4. Selection Process

Two reviewers independently screened titles and abstracts and assessed full-text articles for eligibility; disagreements were resolved through consultation with a third reviewer. No automation tools were used during screening.

### 2.5. Data Extraction

Data extraction was conducted independently by two reviewers using a standardized table; as all items were numeric or explicitly reported, no discrepancies arose. No automation tools were used. From each eligible study, the data extracted included title, authors, and year of publication; study design, sample size, and study duration. The reported findings in the revised research studies were included for this manuscript if they included cognitive outcomes, even if this was not the primary outcome for a given study.

### 2.6. Synthesis Methods

Because of substantial heterogeneity in study designs, comparator conditions, intervention characteristics, and cognitive outcome measures, calculation of a pooled effect estimate was not considered appropriate. The findings were therefore examined through a structured narrative synthesis based on the cognitive changes and between-group differences reported in the included studies.

Reports were organized according to their principal comparison: combined cognitive remediation and physical exercise (CR + PE) versus CR alone, CR + PE versus PE alone, and three-arm designs comparing CR + PE with the individual interventions. Within these groups, findings were summarized for global cognitive composite scores and for the individual cognitive domains assessed by the included studies.

The comparisons were interpreted according to the evidence permitted by each design. Two-arm comparisons could indicate whether adding one intervention provided an incremental benefit relative to the available comparator but could not, by themselves, establish additivity. Three-arm designs including CR + PE, CR alone, and PE alone provided the most direct evidence concerning whether the combined intervention performed better than both interventions delivered separately. This grouping was used to structure the narrative synthesis and did not imply that every included report was capable of directly testing additivity. Synergy, which would require a formal statistical interaction analysis, was not formally evaluated.

### 2.7. Risk-of-Bias Assessment

Risk of bias was assessed at the report level using design-appropriate tools. Randomized controlled trials were evaluated using the Cochrane Risk of Bias 2 (RoB 2) tool. Non-randomized studies were evaluated using the Risk of Bias in Non-Randomized Studies of Interventions (ROBINS-I) tool. Domain-level judgments and an overall risk-of-bias judgment were assigned to each report (see Supplementary Material File S2).

## 3. Results

### 3.1. Study Selection

**Figure 1:**
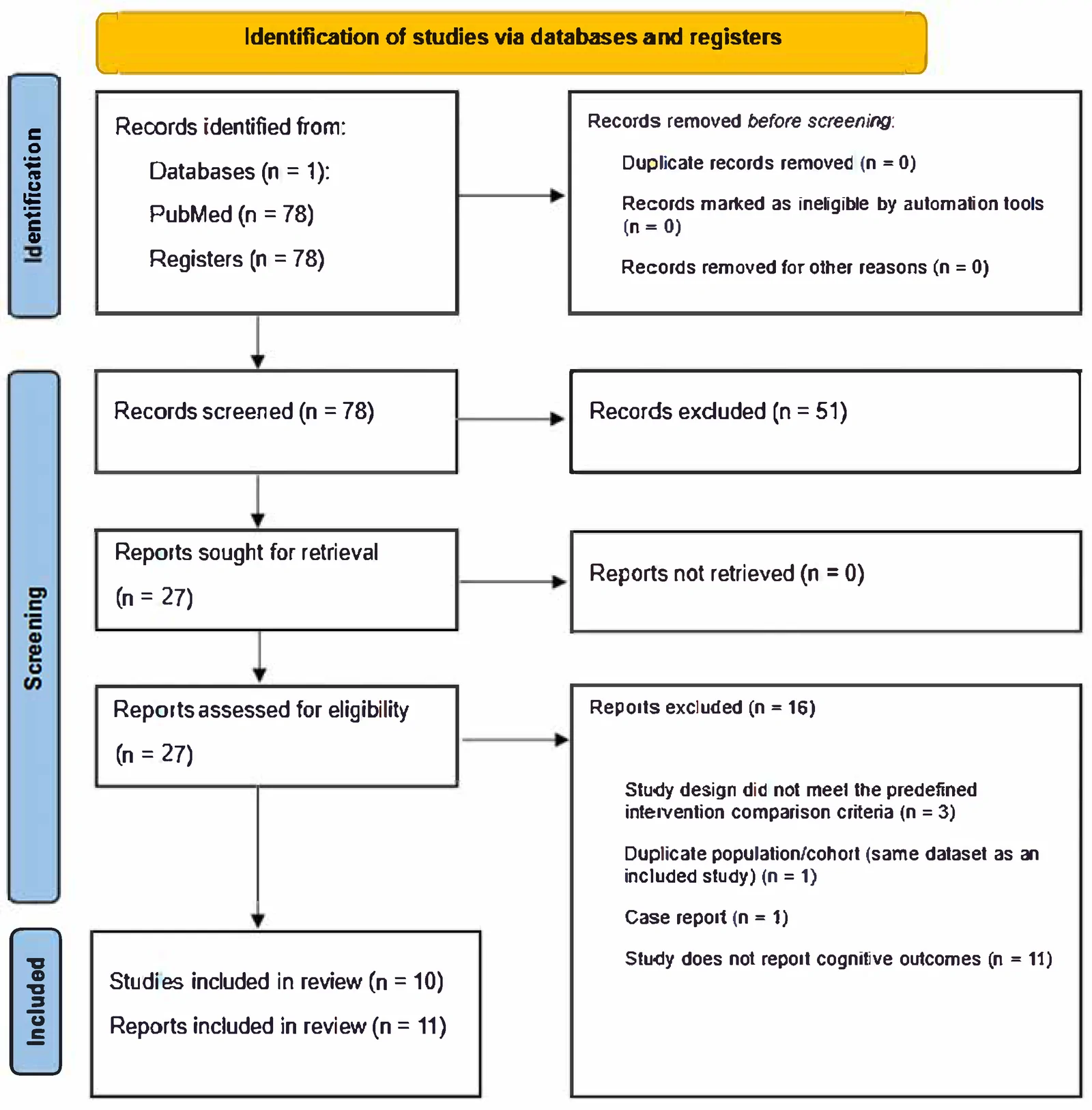
PRISMA flowchart.

The PubMed search identified 78 records, all of which underwent title and abstract screening; no duplicates were identified. Fifty-one records were excluded at this stage, and 27 reports were sought for retrieval, all of which were found and went for full-text eligibility assessment. Of these, 11 reports representing 10 underlying studies met the eligibility criteria and were included in the synthesis. Takahashi et al. [18] and Malchow et al. [19] reported different outcomes from the same underlying trial cohort. The characteristics of the in-cluded reports are summarized in Table 1. The PRISMA 2020 flow diagram summa-rizing study identification, screening, eligibility assessment, and inclusion is shown in Figure 1.

### 3.2. Combined CR and PE Versus CR Alone

Nuechterlein et al. studied the impact of a 6-month program of cognitive training & exercise (CT&E) compared to cognitive training alone (CT) in 47 first-episode schizophrenia outpatients [20]. For the MATRICS Consensus Cognitive Battery (MCCB) overall composite score, significant improvement was found in the CT&E group compared to the CT-only group (F = 3.33, *p* = 0.04). During the first 3 months, cognitive improvement was substantially greater in the CT&E group (mean gain of 6.5 T-score points compared to 2.2 in the CT group; *p* = 0.012, Cohen’s f = 0.43). In the CT&E group, the baseline-covaried MCCB score went from 22.9 (0.9) at baseline to 29.4 (1.0) at 3 months and 29.7 (1.0) at 6 months. In the CT group, the baseline-covaried MCCB score went from 22.9 (0.9) at baseline to 25.1 (1.0) at 3 months and 28.1 (1.0) at 6 months. Improvement in muscular endurance over three months (measured by the number of sit-ups) showed a trend toward correlating with cognitive improvement at three months (MCCB overall composite score: r = 0.28, *n* = 40, *p* < 0.08). In a previous pilot study, the same group of authors [14] found a notable effect on the MATRICS (MCCB) global composite score, where the CT&E group showed improvement with a large effect size compared to the CT-only group (Cohen’s f = 0.48). When exploring this in several cognitive domains, considerable differences were found for social cognition (Cohen’s f = 0.65) and working memory (Cohen’s f = 0.50), and moderate differences were found for processing speed (Cohen’s f = 0.38) and attention/vigilance (Cohen’s f = 0.33).

In a randomized controlled trial of exercise on augmenting the effects of cognitive remediation in persons with severe mental illness, McGurk et al. explored whether adding a 30 h aerobic exercise program over 10 weeks to an equally intensive cognitive remediation program (CR + E) improved cognitive functioning more than cognitive remediation alone (CR-Only). Thirty-four participants with schizophrenia or bipolar disorder were randomly assigned to CR + E or CR-Only, and cognitive functioning was assessed at baseline and post-treatment [21]. Both groups (CR + E and CR-Only) showed significant improvement in global cognitive function as measured by the MATRICS Consensus Cognitive Battery (MCCB), but there were no significant differences between the groups. The cognitive composite score (MCCB) for the CR + E group improved from 33.85 (SD = 6.64) to 40.15 (SD = 5.01), while the CR-Only group improved from 34.06 (SD = 8.01) to 42.66 (SD = 3.15). However, on the Trail Making Test Part B, both groups showed an increase in response time (indicating a decline in performance), although the trend suggests that the CR + E group experienced less deterioration compared to the CR-Only group (*p* = 0.099, η^2^ = 0.10).

A German group led by Oertel-Knöchel studied two disease groups (*n* = 22 major depression patients; *n* = 29 schizophrenia patients) that were matched for age, gender, duration of disease, and years of education and received cognitive training combined either with aerobic physical exercise or with mental relaxation training [22]. They found an improvement in speed of processing in patients with schizophrenia (F(46) = 37.55, *p* < 0.001) but no significant intervention effect (*p* > 0.05). However, the effect size was larger in the group that combined cognitive training with exercise (d = 0.57), in comparison with the relaxation group (d = 0.43) and the control group (d = 0.50). In working memory, a significant improvement was observed in patients with schizophrenia (F(46) = 9.34, *p* < 0.05), with a significant intervention group effect (F(46) = 10.23, *p* = 0.02) and a significant time-by-intervention effect in the group that combined cognitive remediation with aerobic exercise (t = 18.82, *p* < 0.001). Visual learning improved in both major depression and schizophrenia patients. The effect size was larger in the group that combined cognitive remediation with exercise (d = 0.91), whereas it was moderate in the group that combined cognitive remediation with relaxation (d = 0.47) and small in the control group (d = 0.25) [22].

Takahashi et al. investigated 23 healthy controls (HC-EX group), 21 schizophrenia patients who performed aerobic exercise (SCZ-EX group), and 21 schizophrenia patients who played table soccer (SCZ-EXCONT group). The 12-week exercise intervention was combined with computer-assisted cognitive remediation training from week 6 to week 12 [18]. In executive function, they found that TMT-B completion time in the SCZ-EX group showed no significant changes (Pre = 65.3 s, Post = 63.2 s, *p* = 0.917). Conversely, in the SCZ-EXCONT group, TMT-B completion time improved significantly after the intervention (Pre = 81.1 s, Post = 70.2 s, *p* = 0.039). In processing speed, no significant differences were found in TMT-A completion time in the SCZ-EX group (Pre = 30.3 s, Post = 30.2 s, *p* = 0.626). In the SCZ-EXCONT group, there was a significant improvement in TMT-A performance following the intervention (Pre = 34.1 s, Post = 28.4 s, *p* = 0.001). In terms of short-term memory, no significant differences were found in short-term memory scores in the SCZ-EX group (Pre = 13.5, Post = 13.8, *p* = 0.681). In the SCZ-EXCONT group, there was a significant improvement in scores following the intervention (Pre = 12, Post = 13.6, *p* = 0.047).

Malchow et al. evaluated the effects of an enriched environment paradigm consisting of bicycle ergometer training and add-on computer-assisted cognitive remediation training [19]. For short-term memory (STM), significant effects of time (F = 4.6; df = 2, 53; *p* = 0.015) and significant time × group interactions (F = 2.7; df = 4, 108; *p* = 0.034) were observed. For long-term memory (LTM), a significant effect of time was found (F = 11.0; df = 2, 53; *p* < 0.0005). The ET + CR improved from 21.9 ± 5.6 to +12.7% at 3 months vs. 6 weeks (*p* = 0.030). No improvements were observed in the CR group. Significant improvement was observed in the total score of the Wisconsin Card Sorting Test (WCST) in the ET + CR group (baseline score: 34.5 ± 6.8), with improvement also observed at 3 months compared to 6 weeks (Z = −2.6, *p* = 0.008) but no significant improvements in the CR group.

### 3.3. Combined CR and PE Versus PE Alone

Dai et al. recruited a group of ninety-six patients with schizophrenia and cognitive impairment, who were randomly assigned to the control, aerobic exercise (AE), and computerized cognitive remediation therapy combined with aerobic exercise (CAE) groups [23]. Processing speed was assessed using the Trail Making Test-A (TMT-A) and the Symbol Coding Test (SCT). No significant differences were found among the three groups during the first 4 weeks. At 8 weeks, a significant overall difference among the groups was found for the TMT-A (F = 5.768, *p* = 0.005, η^2^ = 0.127) and the SCT (F = 5.148, *p* = 0.008, η^2^ = 0.115). The CAE group showed significant improvements compared to the AE group (TMT-A: *p* = 0.04; SCT: *p* = 0.008) and the control group (TMT-A: *p* = 0.002; SCT: *p* < 0.001). No significant differences were found between the AE group and the control group. Changes from baseline to week 8 differed significantly among the three groups (TMT-A: F = 7.937, *p* < 0.001, η^2^ = 0.167; SCT: F = 8.882, *p* < 0.001, η^2^ = 0.184), confirming that CAE showed the greatest improvement in processing speed.

Cognitive flexibility was assessed using the Stroop Word Test (SWT) and Stroop Colour Test (SCoT). No significant differences were found among the three groups during the first 4 weeks. At 8 weeks, a significant overall difference was found among the groups in the SWT (F = 3.657, *p* = 0.030, η^2^ = 0.085) and SCoT (F = 5.300, *p* = 0.007, η^2^ = 0.118), indicating that the groups evolved differently. At 8 weeks, the CAE group showed significant improvement in both tests compared to the AE group (SWT: *p* = 0.009; SCoT: *p* = 0.033) and the control group (SWT: *p* < 0.001; SCoT: *p* = 0.001). Again, no significant differences were observed between the AE group and the control group. Changes from baseline to week 8 were significantly different among the three groups (SWT: F = 9.934, *p* < 0.001, η^2^ = 0.201; SCoT: F = 7.514, *p* = 0.001, η^2^ = 0.160), with CAE showing the most pronounced improvements. At 12 weeks, improvements in processing speed and cognitive flexibility were maintained in the CAE group, with significant differences compared to the other groups and no significant differences between the AE and control groups [23].

A French group led by Dubreucq et al. conducted a quasi-experimental study evaluating the effects of an exercise-enriched, integrated social cognitive remediation (SCR) intervention, called Remed Rugby (RR), compared with an active control group practicing Touch Rugby (TR) [24]. Moderate improvements in verbal abstraction were observed in the RR group compared to the TR group (*p* = 0.008, d = 0.554). Additionally, significant improvements were found in immediate memory (BEMRI.NB, *p* = 0.008) and delayed memory (BEMRD.NB, *p* = 0.021) in the RR group. Delayed memory showed improvement post-treatment (*p* = 0.036), whereas immediate memory did not show a significant change post-treatment (*p* = 0.158). Furthermore, improvements were found in cognitive flexibility and processing speed, reflected in reduced completion times on the Trail Making Test A (*p* = 0.006 post-treatment, *p* = 0.001 at follow-up, *p* = 0.02 post-treatment change) and Trail Making Test B (*p* = 0.043 post-treatment, *p* = 0.008 at follow-up, *p* = 0.04 post-treatment change). Also, a significant improvement was observed in information encoding—assessed via the WAIS-IV Coding subscale—with statistically significant differences post-treatment (*p* < 0.001) and at follow-up (*p* < 0.001). Significant improvement was evident at the post-treatment follow-up (*p* = 0.015).

### 3.4. Combined Cognitive Remediation and Physical Exercise in a Three-Arm Design

Shimada et al. studied the effect of aerobic exercise (AE) versus cognitive remediation (CR) versus a combination of both (AE + CR) among patients with schizophrenia in a three-arm, randomized controlled study [25]. They found that the group that combined CR and AE showed significantly greater improvement than both CR alone and AE alone. The adjusted difference was 0.98 versus EA (95% CI: 0.14 to 1.82; *p* = 0.017) and 0.90 versus CR (95% CI: 0.13 to 1.68; *p* = 0.019). There were no differences between the CR and AE groups (estimate = −0.08; 95% CI: −0.79 to 0.63; *p* = 0.831). In verbal memory, a greater improvement was observed in the AE + CR group compared to AE and CR alone. The adjusted difference in change was significant versus AE alone, with an estimate of 0.79 (95% CI: 0.31 to 1.27; *p* < 0.001), and versus CR alone, with an estimate of 0.44 (95% CI: 0.00 to 0.88; *p* = 0.048). The comparison between CR and AE did not reach significance, although it showed a trend favoring CR (estimate = −0.35; 95% CI: −0.75 to 0.06; *p* = 0.090). In working memory, the combined group showed significantly greater improvement than the cognitive remediation-only group, with an adjusted difference of 0.97 (95% CI: 0.35 to 1.59; *p* = 0.001). Comparison against aerobic exercise alone showed a trend favoring the combined treatment, though without statistical significance (estimate = 0.43; 95% CI: −0.08 to 0.95; *p* = 0.096). A trend favoring AE over CR was also observed, without reaching significance (estimate = 0.54; 95% CI: −0.08 to 1.15; *p* = 0.096).

In verbal fluency, absolute improvements were observed in all three groups, with the greatest numerical gain in the combined intervention. However, there were no significant differences between the groups. All three groups also showed numerical improvements in motor speed, but no significant differences were observed between the interventions. CR showed the greatest absolute change, although this did not translate into statistical superiority. In attention and processing speed, the combined intervention showed significantly greater improvement than CR alone, with an adjusted difference of 0.93 (95% CI: 0.14 to 1.71; *p* = 0.016). Compared with aerobic exercise alone, a trend favoring the combined treatment was observed, though it did not reach statistical significance (estimate = 0.69; 95% CI: −0.05 to 1.43; *p* = 0.071). There were no differences between AE and CR (estimate = 0.23; 95% CI: −0.44 to 0.90; *p* = 0.490). For executive function, the combination of AE and CR showed significantly greater improvement than either treatment by itself. Compared with AE, the adjusted difference was 1.22 (95% CI: 0.35 to 2.10; *p* = 0.004), whereas compared with CR, it was 1.28 (95% CI: 0.35 to 2.21; *p* = 0.004). There were no differences between AE and CR (estimate = 0.05; 95% CI: −0.74 to 0.85; *p* = 0.892) [25].

However, another study exploring possible synergistic benefits of physical and cognitive exercise in schizophrenia found some results favoring physical exercise alone [26]. In particular, a significant group effect was found for processing speed (Processing Speed Index—PSI) (F [2, 81] = 14.78, *p* < 0.001), with a significant improvement in the physical exercise (PE) group compared to the cognitive training (CT) and combined PE + CT groups (*p* < 0.01). No significant differences were observed between the CT and PE + CT groups. The PE group, with a baseline score of 74.34 ± 11.84, showed an increase of approximately 10 points in PSI, reaching a value close to 85 post-intervention. The CT group, with a baseline score of 72.61 ± 12.35, showed an increase of around 4–5 points, reaching a level close to 77 post-intervention. The PE + CT group, with a baseline score of 72.88 ± 11.22, exhibited an increase of approximately 6 points, with a post-intervention value close to 79. Another significant group effect was found for working memory (Working Memory Index—WMI) (F [2, 81] = 11.40, *p* < 0.01), where both the PE and PE + CT groups showed significant improvement compared to the CT-only group (*p* < 0.001). The PE group started with a baseline value of 83.63 ± 9.05, which increased by approximately 4 points, reaching values close to 88 post-intervention. The CT group, with a baseline score of 81.48 ± 10.21, showed an increase of around 3 points, reaching a level close to 84 post-intervention. The PE + CT group, with a baseline score of 81.77 ± 8.97, showed an increase of nearly 6 points, with post-intervention values around 87.

However, at the 2-month follow-up, only the PE + CT group sustained improvements in working memory and processing speed, with a significant group effect (F [3, 78] = 22.19–23.81, *p* < 0.001). In contrast, improvements in the PE and CT groups partially diminished, with scores returning to levels close to baseline. The PE + CT group at the 2-month follow-up maintained greater stability in improvements, with PSI values around 80 and WMI values near 87. This study found that participants with high motivation showed greater improvements in processing speed and working memory across all groups. The relationship between initial motivation and change in WMI was significant in the CT group (r = 0.22, *p* < 0.05) and the PE + CT group (r = 0.26, *p* < 0.01) [26].

Agostoni et al. carried out a randomized, multicenter, single-blind clinical trial with three parallel arms: cognitive remediation (CR), aerobic exercise (AE), and cognitive remediation plus aerobic exercise (CR + AE). The combined intervention produced significantly greater improvements than CR alone in attention, working memory, and verbal learning and greater improvements than AE alone in processing speed, working memory, and verbal learning. The authors concluded that “Two is Better Than One” [27].

### 3.5. Risk of Bias

Risk of bias was assessed in all 11 included reports. Among the seven randomized reports, four received an overall risk-of-bias judgment of “some concerns” [18,20,21,26], while three were judged to be at high risk [19,22,27]. The most consistent source of concern was the selection of the reported result, as the available information was insufficient to exclude selection among multiple eligible outcomes or analyses. The high-risk judgments were driven mainly by limitations related to missing outcome data. Bias due to deviations from intended interventions was judged to be low in all seven randomized reports, while bias in outcome measurement was low in six and raised some concerns in only one [18]. All four non-randomized reports were judged to be at serious risk of bias according to ROBINS-I criteria [14,23,24,25], principally because of potential confounding.

## 4. Discussion

This systematic review addresses a clinically relevant question: Is the combination of physical exercise and cognitive remediation better than each of these interventions by itself? This will contribute to a broader question, which is how to appropriately treat cognitive symptoms in patients with schizophrenia, considering the information currently available. This is important, considering the clinical relevance of cognitive symptoms for community functioning and quality of life, particularly since usual pharmacological treatments for schizophrenia lack significant effects for improving cognitive symptoms.

Cognitive impairment in primary psychotic disorders is ubiquitous, with approximately 80% of patients exhibiting clinically significant impairment. However, even in the absence of clinically significant cognitive impairment, it has been argued that all individuals with a primary psychotic disorder perform at a level below what would be expected had they never developed a psychotic illness [10]. Therefore, helping these patients could have a significant impact on public health.

After reviewing all selected papers, in most cases, the combined intervention was superior to both cognitive remediation and physical exercise as individual interventions. This was the case for composite scores but also for several cognitive domains, such as verbal memory, executive function, working memory, and attention/processing speed. In several studies, it was not clear whether physical exercise or cognitive remediation was better as individual interventions. In most cases, they produced comparable cognitive improvements, with no significant differences between them in any domain. However, it was clear that the combination was better [26].

Potential shared molecular mechanisms must be considered, as both cognitive remediation and physical exercise can increase the expression of brain-derived neurotrophic factor (BDNF) [16]. As Jahshan et al. have explained [28], given that cognitive remediation may depend on intact neuroplasticity to produce cognitive gains, it is reasonable to combine it with strategies that harness patients’ neuroplastic potential. Future reviews could deepen the investigation into how the combination of physical exercise and cognitive remediation may have additive effects in the increase in BDNF levels or other biological markers at the molecular, neurophysiological, or neuroimaging levels.

This review has several strengths. It provides an updated synthesis focused specifically on the potential additive cognitive effects of combining cognitive remediation and physical exercise in people with schizophrenia spectrum disorders. The review followed a registered protocol, applied explicit eligibility criteria and PRISMA-based study selection, and incorporated recent evidence, including two three-arm randomized trials published in 2025. Methodological quality was evaluated at the report level using design-specific risk-of-bias tools, with detailed assessments provided in the Supplementary Materials. The synthesis also distinguished between three-arm designs capable of directly examining additivity and studies providing comparator-specific or indirect evidence.

Several limitations should nevertheless be acknowledged. The literature search was restricted to PubMed, and publication bias was not formally assessed; therefore, relevant studies or selectively unpublished findings may have been missed. The included reports differed substantially in design, patient characteristics, intervention content and duration, comparator conditions, and the cognitive domains and instruments used to assess outcomes. This heterogeneity precluded a meaningful meta-analysis, necessitated a narrative synthesis, and limited the comparability and generalizability of the findings. Furthermore, only a subset of the included studies directly compared CR + PE with both CR alone and PE alone. Much of the available evidence can therefore demonstrate only an incremental benefit relative to a particular comparator rather than definitive evidence of additivity.

The evidence was also affected by a variable risk of bias. Among the seven randomized reports, four received an overall judgment of “some concerns,” while three were judged to be at high risk. All four non-randomized reports were judged to be at serious risk, principally because of potential confounding. Antipsychotic treatment was not consistently accounted for and may have influenced cognitive performance and participation in physical exercise. The findings should therefore be interpreted cautiously and considered suggestive rather than definitive.

Despite these limitations, the results summarized in this review may help researchers design future studies aimed at determining which characteristics of physical activity, including exercise type, duration, and frequency, are best suited to particular forms of cognitive remediation, such as compensatory or restorative approaches targeting executive or perceptual functioning. Another important direction is the identification of clinical indicators or biomarkers that could help select the most appropriate combination of exercise and cognitive remediation for each patient, supporting a more personalized approach.

This systematic review could also be useful for policymakers and clinicians who are involved in defining future treatment guidelines. Current practice should match up-to-date knowledge, and a better understanding of the additive effects of cognitive remediation and physical exercise for improving cognition in schizophrenia could positively impact the lives of many patients suffering from cognitive symptoms.

## Figures and Tables

**Table 1:** Characteristics of the included studies.

Author	Country	Design	Participants	Exercise Methods	Cognitive Remediation Methods	Assessment Instruments	Main Results
Nuechterlein et al. (2016) [14]	United States	Controlled, prospective, two-group feasibility pilot study. Cognitive training plus aerobic exercise, CT&E; Cognitive training alone, CT.	16 patients with recent-onset schizophrenia: CT: *n* = 9; CT&E: *n* = 7. Mean time from onset of psychosis to program entry: 9.2 +/− 6.6 months.	10-week aerobic exercise and calisthenics program. Applied only to the CT&E group. Total dose: 150 min per week; distributed over 4 days per week. Frequency: 2 clinic sessions of 45 min; 2 home sessions of 30 min.	Both groups received the same Posit Science computerized cognitive training program. Duration: 10 weeks. Frequency: 2 days per week; 2 h per day; 4 h per week in total. Total dose: 20 h of neurocognitive training; 20 h of social cognition training. First 5 weeks: neurocognitive training with the Brain Fitness Program. Second 5 weeks: social-cognitive training with the Targeted Affect Remediation Application, TARA.	**(1) Cognition** MATRICS Consensus Cognitive Battery, MCCB. **(2) Functioning** Role Functioning Scale, RFS. **(3) Biomarkers** Serum BDNF, measured by ELISA in a subsample. **(4) Clinical symptoms** Brief Psychiatric Rating Scale, BPRS, used to characterize baseline clinical stability.	The CT&E group showed greater improvement than CT in the MCCB global composite score: Cohen f = 0.48, a large effect size. The domains with the largest differential gains favoring CT&E were: social cognition; working memory; processing speed; attention/vigilance. In functioning, CT&E showed greater improvement than CT in: independent living, a very large effect; family relationships; work productivity. In a subsample of 4 CT&E participants, serum BDNF increased: mean change +4220 +/− 4110 pg/mL.
Nuechterlein et al. (2023) [20]	United States	Randomized controlled trial, two groups, with intention-to-treat analysis. Two parallel groups: Cognitive training plus exercise, CT&E; Cognitive training alone, CT.	47 outpatients with a first episode of schizophrenia or related disorders. CT&E: *n* = 24. CT: *n* = 23. Mean time since onset of psychosis: 8.9 months.	Progressive 24-week aerobic exercise and physical conditioning program. Frequency: 2 clinic sessions per week; 2 home sessions per week. Overall target: 150 min per week of moderate aerobic activity.	Posit Science computerized cognitive training, common to both groups. Frequency: 2 h per day, 2 days per week. Weekly dose: 4 h. Total duration: 24 weeks. First 12 weeks: Neurocognitive training with BrainHQ; 48 h in total. Second 12 weeks: Social cognition training with SocialVille; 48 h in total.	**(1) Cognition** MATRICS Consensus Cognitive Battery, MCCB. **(2) Functioning** Global Functioning Scale. **(3) Biomarkers** Serum BDNF, measured by ELISA. **(4) Clinical symptoms** Brief Psychiatric Rating Scale, BPRS.	The MCCB global composite score showed a significant group x time interaction over the 6 months: F = 3.33; *p* = 0.04, favoring CT&E. During the first 3 months, cognitive improvement was significantly greater in CT&E than in CT. Work and school functioning improved significantly more with CT&E. Social functioning improved in both groups, but without a significant between-group difference. BDNF increased over time in both groups. Cognitive improvement at 3 months predicted improvement in work and school functioning at 6 months. The amount of exercise completed was associated with greater benefits, both cognitive improvement and work and school improvement.
McGurk et al. (2021) [21]	United States	Randomized controlled trial with two parallel groups. Intervention duration: 10 weeks. Groups: Cognitive remediation plus aerobic exercise, CR + E; Cognitive remediation alone, CR-Only.	34 participants with serious mental illness: CR + E: *n* = 17; CR-Only: *n* = 17. Diagnoses included according to DSM-5: schizophrenia; schizoaffective disorder; schizophreniform disorder; bipolar I disorder; bipolar II disorder. Proportion with a schizophrenia spectrum diagnosis: approximately 65%.	Supervised aerobic exercise program over 10 weeks. Applied exclusively to the CR + E group. Frequency: 3 sessions per week. Duration: 50 minu per session.	Both groups received computerized cognitive remediation with COGPACK, version 8.0. Total duration: up to 30 h. Frequency: 1 hour per session; 3 times per week; over 10 weeks.	**(1) Cognition** MATRICS Consensus Cognitive Battery, MCCB, adapted. **(2) Biomarkers** Total serum BDNF. Mature BDNF. Mature/total BDNF ratio. Methods: Aviscera-Bioscience ELISA for mature BDNF; Quantikine Total BDNF Immunoassay for total BDNF. **(3) Clinical symptoms** Expanded Brief Psychiatric Rating Scale, BPRS. Montgomery-Asberg Depression Rating Scale, MADRS.	**Global cognition** Both groups improved significantly in the cognitive composite score: main effect of time: *p* < 0.001. There was no significant group x time interaction. **Domain-level results** Significant effects of time, with improvement in both groups, were observed in 8 of the 11 individual tests: TMT-A: *p* < 0.001. Symbol Coding: *p* = 0.021. HVLT verbal learning: *p* = 0.003. Letter-Number Span: *p* = 0.049. BVMT visual learning: *p* = 0.006. Delayed visual memory: *p* = 0.036. Category fluency: *p* = 0.004. Attention on the CPT: *p* = 0.008. **Between-group comparison** No group x time interaction was statistically significant. Only a trend favoring CR + E was observed on TMT-B: group x time interaction: *p* = 0.099. **BDNF** No differences were observed between CR + E and CR-Only. There were no significant effects of: group; time; group x time interaction.
Oertel-Knochel et al. (2014) [22]	Germany.	Randomized controlled trial with three parallel groups, including inpatients with schizophrenia and major depressive disorder. Main groups: Cognitive training plus aerobic exercise. Cognitive training plus mental relaxation. Waiting list without intervention.	51 inpatients completed the study: Schizophrenia: *n* = 29; Major depressive disorder: *n* = 22. Distribution of the main sample: Exercise: *n* = 16; Relaxation: *n* = 17; Waiting list: *n* = 18. Distribution of patients with schizophrenia: Exercise: *n* = 8; Relaxation: *n* = 11; Waiting list: *n* = 10.	Group aerobic exercise program combined with circuit training. Frequency: 3 sessions per week. Duration: 45 min per session; over 4 weeks; 12 sessions in total.	Both active groups received a combined program based on COGPACK. Frequency: 3 sessions per week; 30 min per session; over 4 weeks. Total dose: 12 sessions; approximately 6 h of cognitive training.	**(1) Cognition** MATRICS-based battery. **Clinical symptoms and quality of life** State-Trait Anxiety Inventory, STAI, state anxiety subscale. SF-12, mental health subscale, for subjective perception of psychological well-being. **In schizophrenia:** Positive and Negative Syndrome Scale, PANSS; Revised Hallucination Scale, RHS. **In major depression:** Beck Depression Inventory-II.	**Processing speed** A significant improvement was observed between pre- and post-treatment: main effect of time: *p* < 0.001. There was no significant effect of intervention group and no diagnosis x intervention x time interaction. **Working memory** Significant effects were found for: time; diagnosis; intervention group. There was a significant interaction: intervention x diagnosis x time *p* < 0.01. Post hoc analysis showed a greater improvement in working memory in the patients with schizophrenia of the exercise group: intervention x time interaction: *p* < 0.001. **Visual learning** A significant improvement was observed: *p* = 0.004. There was also an effect of diagnosis *p* = 0.01. Patients with schizophrenia had lower baseline scores and greater subsequent improvement. **Verbal learning** There were no significant effects of: time; diagnosis; intervention group; interactions. Verbal learning did not improve significantly under any condition. **Cognitive effect sizes** Exercise group: processing speed, working memory and verbal learning: d = 0.47–0.57, moderate effects; visual learning: d = 0.91, large effect. Relaxation group: processing speed and visual learning: d = 0.43–0.47; working memory and verbal learning: d = 0.18–0.24. Waiting list: processing speed: d = 0.50; remaining domains: d = 0.23–0.35. **Schizophrenia symptoms** Positive symptoms: no significant changes were observed. Negative symptoms: significant effect of the intervention: *p* = 0.02; group x time interaction: *p* = 0.04. Negative symptoms decreased in both: exercise plus cognitive training; relaxation plus cognitive training.
Takahashi et al. (2020) [18]	Germany.	Longitudinal, controlled, non-randomized study with three parallel groups. Groups: Patients with schizophrenia undergoing aerobic exercise, SCZ-EX. Patients with schizophrenia who played table soccer as an active control, SCZ-EXCONT. Healthy controls undergoing aerobic exercise, HC-EX.	65 participants: SCZ-EX: *n* = 21; SCZ-EXCONT: *n* = 21; HC-EX: *n* = 23.	Aerobic training on a cycle ergometer. Duration: 12 weeks. Frequency: 3 sessions per week. Session duration: 30 min. **Active control** The SCZ-EXCONT group played table soccer: 30 min; 3 times per week; over 12 weeks.	Computerized COGPACK program, versions 8.19 D/8.30 DE. Administered to all three groups: SCZ-EX; SCZ-EXCONT; HC-EX. Start: after the first 6 weeks of exercise or table soccer. Duration: weeks 6 to 12; 6 weeks in total. Frequency: 2 sessions per week. Duration: 30 min per session.	**(1) Neuroimaging** 3-tesla structural magnetic resonance imaging. T1 MPRAGE sequence. Processing with FreeSurfer 6.0. Regions of interest: left and right entorhinal cortex; left and right parahippocampal cortex; left and right lateral prefrontal cortex; left and right medial prefrontal cortex. **(2) Functioning and social adjustment** Global Assessment of Functioning, GAF. Social Adjustment Scale-II, SAS-II. **(3) Clinical symptoms** Positive and Negative Syndrome Scale, PANSS. Clinical Global Impression-Severity, CGI-S. Calgary Depression Scale for Schizophrenia, CDSS. **(4) Cognition** Verbal Learning and Memory Test, VLMT. Trail Making Test A, TMT-A. Trail Making Test B, TMT-B. Wisconsin Card Sorting Test, WCST.	**Cortical thickness** The SCZ-EX group showed a significant increase in the thickness of the right entorhinal cortex at week 6; the increase was not maintained. No significant longitudinal changes were observed in: left entorhinal cortex; parahippocampal cortex; lateral prefrontal cortex; medial prefrontal cortex. The SCZ-EXCONT and HC-EX groups showed no significant changes in cortical thickness after correction for multiple comparisons. **Prediction of social adjustment** In SCZ-EX, a greater baseline thickness of the right lateral prefrontal cortex was associated with a greater improvement in overall social adjustment: *p* = 0.005. No significant associations were found between baseline cortical thickness and: GAF; SAS-II social/leisure; cognitive outcomes. No equivalent correlations were found in SCZ-EXCONT or HC-EX. **Global functioning** The SCZ-EX group showed a significant improvement in GAF. SCZ-EXCONT showed no change. **Social adjustment** In SCZ-EX the following improved: SAS-II social/leisure: *p* = 0.006; SAS-II overall: *p* = 0.003. There was no significant improvement in the work domain. The SCZ-EXCONT group showed no significant changes in the social adjustment subscales. **Cognition** SCZ-EX showed no significant improvements. SCZ-EXCONT showed an improvement in TMT-A: *p* = 0.001. The improvement in short-term memory and TMT-B in the table soccer group did not reach the corrected threshold. **Clinical symptoms** In SCZ-EX there were no significant changes in: PANSS; CGI-S; CDSS. SCZ-EXCONT showed nominal reductions in: PANSS positive: *p* = 0.040; PANSS total: *p* = 0.014; but these did not exceed the corrected threshold of *p* < 0.00833.
Malchow et al. (2015) [19]	Germany.	Longitudinal, controlled, single-center, non-randomized study with three parallel groups. Groups: Patients with schizophrenia undergoing endurance training plus cognitive remediation. Patients with schizophrenia who played table soccer plus cognitive remediation. Healthy controls undergoing endurance training plus cognitive remediation.	64 patients with schizophrenia and 36 healthy controls were initially assessed. Sample included in the main analyses: Schizophrenia + endurance training: *n* = 22; Schizophrenia + table soccer: *n* = 21; Healthy controls + endurance training: *n* = 23. Total analyzed: 66 participants, although some analyses used 65 because of missing data.	Continuous endurance training on a cycle ergometer. Duration: 3 months. Frequency: 3 sessions per week. Duration: 30 min per session. **Active control** The control group played table soccer: 30 min; 3 times per week; over 3 months.	Computerized COGPACK program, versions 8.19 D/8.30 DE. Administered to all three groups. Start: after the first 6 weeks of exercise or table soccer. Duration: 6 weeks. Frequency: 2 sessions per week. Duration: 30 min per session. Approximate total dose: 12 sessions; 6 h of training.	**(1) Global functioning** Global Assessment of Functioning, GAF. **(2) Social adjustment** Social Adjustment Scale-II, SAS-II. **(3) Clinical symptoms** Positive and Negative Syndrome Scale, PANSS. Clinical Global Impression-Severity, CGI-S. Calgary Depression Scale for Schizophrenia, CDSS. **(4) Cognition** Verbal Learning Memory Test, VLMT. Trail Making Test A, TMT-A. Trail Making Test B, TMT-B. Wisconsin Card Sorting Test, WCST.	**Global functioning** The GAF showed: a significant main effect of time; a time x group interaction. In the exercise plus cognitive remediation group, GAF improved by +16.6% from baseline to month 3. The table soccer plus cognitive remediation group showed no significant improvement. **Social adjustment** In the exercise group the SAS-II overall score decreased, *p* = 0.003. The following also improved: social and leisure activities; household functioning. The table soccer group showed no significant changes. **Clinical symptoms** The following were observed: a main effect of time on PANSS total; a main effect of time on PANSS negative; a time x group interaction on PANSS positive; a time x group interaction on PANSS negative. In the exercise group, negative symptoms decreased. The table soccer group showed reductions in: PANSS positive and PANSS total. **Verbal memory** Short-term memory: main effect of time; time x group interaction. In the exercise group it improved significantly between week 6 and month 3; in the table soccer group it improved significantly between baseline and week 6. Long-term memory: main effect of time; the exercise group improved significantly between week 6 and month 3. The table soccer group showed no significant changes. **Processing speed and attentional set-shifting** TMT-A: main effect of time; time x group interaction. The table soccer group improved significantly from week 6 to month 3 and from baseline to month 3. TMT-B: main effect of time; the table soccer group improved significantly from week 6 to month 3 and from baseline to month 3. The exercise group showed no significant changes on TMT-A or TMT-B. **Cognitive flexibility** In the exercise group, the WCST improved significantly between week 6 and month 3. There was no significant improvement relative to baseline. The table soccer group showed no significant changes.
Dai et al. (2022) [23]	China	Longitudinal, randomized, controlled, single-blind trial with three parallel groups. Treatment as usual, or control group; Aerobic exercise, AE; Computerized cognitive remediation plus aerobic exercise, CAE.	96 inpatients with schizophrenia and cognitive impairment were initially randomized. Fourteen participants withdrew before receiving the interventions: control: 1; CAE: 6; AE: 7. Sample included in the final analysis: 82 participants: control: *n* = 31; CAE: *n* = 26; AE: *n* = 25.	Supervised aerobic exercise program over 8 weeks. Applied to the AE and CAE groups. Frequency: 5 days per week, Monday to Friday. Duration: 45 min per session.	Computerized Cognitive Remediation Therapy, CCRT. Administered only to the CAE group. Duration: 8 weeks. Frequency: 2 sessions per week. Session duration: 30 min. Total dose: 16 sessions; approximately 8 h of training.	**(1) Cognitive screening** Montreal Cognitive Assessment, MoCA, Chinese version. **(2) Processing speed** Trail Making Test, Part A, TMT-A. Brief Assessment of Cognition in Schizophrenia: Symbol Coding Test, SCT. **(3) Cognitive flexibility** Stroop Colour-Word Test, SCWT. **(4) Clinical symptoms** Positive and Negative Syndrome Scale, PANSS. **(5) Biomarkers** Serum BDNF, determined by ELISA.	The combination of CCRT and aerobic exercise produced a significantly greater improvement in processing speed than exercise alone and treatment as usual. **TMT-A, baseline to week 8 change** Comparisons: CAE versus AE: *p* = 0.040; CAE versus control: *p* = 0.002; no differences between AE and control. Symbol Coding Test, baseline to week 8 change: CAE versus AE: *p* = 0.008; CAE versus control: *p* < 0.001; no differences between AE and control. **Cognitive flexibility, week 8** The CAE group was significantly superior to both AE and control on both tests. There were no significant between-group differences in the Stroop Colour-Word condition, which assesses interference inhibition. **Clinical symptoms** The reduction in negative symptoms differed significantly between groups: CAE showed greater improvement than the control group: *p* = 0.015. No significant between-group differences were found in: PANSS positive; general psychopathology; PANSS total. **Serum BDNF** Baseline to week 8 change: Both CAE and AE showed a significantly greater increase than control: both comparisons *p* < 0.001. There were no significant differences between CAE and AE. No significant correlations were found between BDNF changes and cognitive improvements. **Correlations** A greater reduction in negative symptoms was related to better cognitive change.
Dubreucq et al. (2020) [24]	France	Quasi-experimental, controlled, multicenter, prospective, interventional and exploratory trial. Two groups: RemedRugby, RR: integrated social-cognitive remediation intervention enriched with Touch Rugby. Touch Rugby, TR: active control based on collective physical activity alone.	87 clinically stable participants with schizophrenia spectrum disorders according to DSM-5. Distribution: RR: *n* = 57; TR: *n* = 30.	Collective physical activity through Touch Rugby. **RemedRugby group** Twelve weekly 2-hour sessions, held at local rugby clubs. In each session approximately 15 min were devoted to supervised Touch Rugby practice. In addition, there was one specific session focused on Touch Rugby. Participation in three full-day tournaments bringing together patients and professionals from the different centers. **Touch Rugby control group** Twelve 2-hour Touch Rugby sessions. Three full-day tournaments.	RemedRugby program, a manualized and ecological group intervention for social-cognitive remediation. Duration: 12 weekly 2-hour sessions; one introductory session; thematic sessions; one final session; three full-day tournaments. **1. Social-cognitive remediation and social skills training** Training in communication and social problem solving. Role-playing and group feedback. **2. Social-cognitive remediation through a blog** Writing e-mails, questions, comments, compliments and criticism. **3. Cognitive remediation of executive functions** Planning and organization of tournaments. Task allocation and paired work. Problem-solving training. **4. Psychoeducation and stigma reduction** Psychoeducation on stigma and self-stigma. Activities aimed at destigmatization.	**(1) Social functioning** Personal and Social Performance Scale, PSP. Social Cognition-Functional Outcomes Scale, ERF-CS. **(2) Social cognition** Movie for the Assessment of Social Cognition, MASC. PerSo, assessment of social perception and social knowledge. Ambiguous Intentions and Hostility Questionnaire, AIHQ. Questionnaire of Cognitive and Affective Empathy, QCAE. ACSo, self-assessment of social cognition difficulties. **(3) Neurocognition** WAIS-IV Coding, processing speed. WAIS-IV Similarities, verbal abstraction. Trail Making Test A, processing speed. Trail Making Test B, cognitive flexibility. Shopping Test-Revised, planning. BEM-144, immediate and delayed verbal memory. French National Adult Reading Test, f-NART, premorbid intelligence. **(4) Clinical symptoms** Positive and Negative Syndrome Scale, PANSS. **Self-stigma, self-esteem and recovery** Internalized Stigma of Mental Illness Scale, ISMI. Self-Esteem Rating Scale-Short Form, SERS-SF. Boston University Empowerment Scale, BUES. Stage of Recovery Instrument, STORI.	The RR group showed a significantly greater improvement in personal and social functioning than the TR group. The effect on social functioning remained significant at follow-up in the RR group. **Clinical symptoms** RR showed a significantly greater improvement than TR in: PANSS total: *p* < 0.001; PANSS positive: *p* = 0.009; PANSS negative: *p* < 0.001; general psychopathology: *p* < 0.001. Symptom improvements persisted over the 6-month follow-up. The TR control group showed, on average, a worsening of symptoms and social functioning during the intervention period. **Social cognition** RR was superior to TR in the reduction of aggression bias. RR produced a greater improvement in components of social cognition-related disability. RR was superior to TR in verbal abstraction, measured with WAIS-IV Similarities. No significant between-group differences were observed in: processing speed on TMT-A; cognitive flexibility on TMT-B; WAIS-IV Coding; immediate or delayed verbal memory; planning on the Shopping Test. **Self-stigma** RR showed a greater improvement than TR in: stereotype endorsement: *p* = 0.019; discrimination experience: *p* = 0.047.
Shimada et al. (2025) [25]	Japan	Randomized controlled trial with three parallel arms and single blinding. Aerobic exercise, AE; Cognitive remediation, CR; Aerobic exercise plus cognitive remediation, AE + CR.	59 patients with schizophrenia: AE: *n* = 19. CR: *n* = 19. AE + CR: *n* = 21.	Combined individual and group aerobic exercise program, led by occupational therapists. Duration: 12 weeks. Frequency: 2 sessions per week: one individual session; one group session. Duration: 60 min per session.	Computerized cognitive remediation program with RehaCom, Japanese version. Modality: individual computerized sessions; individual verbal sessions aimed at transferring the cognitive gains to everyday functioning. Computerized sessions: twice per week; 60 min per session; 24 sessions; over 12 weeks. Verbal sessions: once per week; 60 min; over 12 weeks.	**(1) Cognition** Brief Assessment of Cognition in Schizophrenia, BACS. **(2) Intrinsic motivation** Three items from the Quality of Life Scale, QLS. **(3) Clinical symptoms** Positive and Negative Syndrome Scale, PANSS. **(4) Functioning** Modified Global Assessment of Functioning for Social Functioning, mGAF-F.	**Cognition** **AE + CR versus AE** The combined intervention produced a significantly greater improvement in: verbal memory; executive function; BACS composite score. There were no significant differences in: working memory; motor speed; verbal fluency; attention. **AE + CR versus CR** The combined intervention was significantly superior in: verbal memory; working memory; attention and processing speed; executive function; BACS composite score. There were no significant differences in: motor speed and verbal fluency. **AE versus CR** No significant differences were observed between AE and CR in any BACS domain or in the composite score. **Intrinsic motivation** No significant between-group differences were observed in QLS score change. **Social functioning** No significant differences were observed in mGAF-F.
Choi et al. (2020) [26]	United States	Randomized community effectiveness study with three parallel groups. Physical exercise alone, PE; Cognitive training alone, CT; Physical exercise plus cognitive training, PE + CT.	85 outpatients with a clinical diagnosis of schizophrenia or schizoaffective disorder. Distribution: PE: *n* = 29; CT: *n* = 27; PE + CT: *n* = 29.	Self-determined, choice-based physical exercise program designed to increase motivation and adherence. At the start of each session participants selected an activity from a menu that included: treadmill; elliptical machine; rowing machine; stationary bicycle; strength exercises on Nautilus machines; free weights; basketball; brisk outdoor walking. PE group: 30 min per session; 3 times per week; 18 h in total. PE + CT group: exercise 30 min; 2 times per week; combined with one weekly CT session; 18 h in total across both interventions.	Cognitive training delivered on Android tablets. Specifically targeted at: processing speed; working memory; visual scanning efficiency. Participants could choose among different tasks and game themes, promoting autonomy and motivation. Difficulty was adjusted individually according to: performance; cognitive load estimated by pupillometry. CT group: 30 min; 3 times per week; 18 h in total. PE + CT group: CT 30 min; once per week; combined with two weekly exercise sessions.	**(1) Cognition** Wechsler Adult Intelligence Scale, Fourth Edition, WAIS-IV: Working Memory Index, WMI; Processing Speed Index, PSI. **(2) Clinical symptoms** Brief Psychiatric Rating Scale, BPRS. **(3) Motivation** Intrinsic Motivation Inventory for Schizophrenia Research, IMI-SR.	Both PE and PE + CT improved working memory significantly more than CT. The PE group showed a significantly greater improvement in processing speed than CT and PE + CT. There was no significant post-treatment difference between CT and PE + CT in processing speed. At follow-up, only the PE + CT group maintained the gains in: working memory; processing speed. Although a pre-post reduction in negative symptoms was observed in PE and PE + CT, there were no significant between-group differences in BPRS change at post-treatment or at follow-up. Analyses by motivation indicated that: participants with higher baseline motivation tended to obtain greater cognitive benefits; participants in the PE + CT group showed improvements in negative symptoms and processing speed regardless of their initial motivation level.
Agostoni et al. (2025) [27]	Italy	Randomized, multicenter, single-blind clinical trial with three parallel arms. Cognitive remediation, CR. Aerobic exercise, AE. Cognitive remediation plus aerobic exercise, CR + AE.	60 patients with a DSM-5 diagnosis of schizophrenia. CR: *n* = 20. AE: *n* = 20. CR + AE: *n* = 20.	Group aerobic exercise program on a stationary bicycle. Applied to: AE; CR + AE. Duration: 3 months. Frequency: 2 sessions per week. Total duration: 1 h per session.	Computerized COGPACK program. Applied to: CR; CR + AE. Duration: 3 months. Frequency: 2 sessions per week. Duration: 1 h per session.	**(1) Cognition** MATRICS Consensus Cognitive Battery, MCCB.	Approximate sample available at post-treatment: CR + AE: 16; AE: 15; CR: 11. Approximate sample available at follow-up: CR + AE: 15; AE: 14; CR: 7. **CR + AE versus CR at post-treatment** The combined intervention produced significantly greater improvements in: attention; working memory; verbal learning. **CR + AE versus AE at post-treatment** The combined intervention was significantly superior in: processing speed; working memory; verbal learning. **Domains without significant post-treatment differences** No significant differences were reported between CR + AE and the single-modality groups in: visual learning; reasoning; social cognition at post-treatment. **Three-month follow-up** In the CR + AE versus CR comparison, differences were reported in: social cognition *p* = 0.01; processing speed *p* = 0.03; result favoring CR. The gains achieved by CR + AE after the intervention remained stable in the remaining domains. In the CR + AE versus AE comparison, no additional significant changes were observed between post-treatment and follow-up; the gains obtained by CR + AE remained stable.

## Data Availability

The original contributions presented in this study are included in the article, and the references can be found in publicly available databases.

## Supplementary Materials

The following supporting information can be downloaded at https://www.mdpi.com/article/10.3390/brainsci16090933/s1, File S1: PRISMA 2020 checklist; File S2: Risk of bias assessments.
