# Supplementary material for: Additive Effects of Cognitive Remediation and Physical Exercise in Patients with Schizophrenia: Cognitive Improvements
Source: Brain Sci. 2026 Aug 31;16(9):933. doi: 10.3390/brainsci16090933 (PMC13604475; doi:10.3390/brainsci16090933)
Supplement: Supplementary file 1 [file brainsci-16-00933-s001.zip › File S2 Risk-of-Bias Assessments]

**Risk-of-Bias Assessments**

# ROBINS-I

## Nuechterlein et al. (2016)

| **ROBINS-I domain** | **Judgment** |
| --- | --- |
| 1. Bias due to confounding | **Serious** |
| 2. Bias in selection of participants into the study | **Low** |
| 3. Bias in classification of interventions | **Low** |
| 4. Bias due to deviations from intended interventions | **Low** |
| 5. Bias due to missing data | **Serious** |
| 6. Bias in measurement of outcomes | **Moderate** |
| 7. Bias in selection of the reported result | **Moderate** |
| **Overall risk of bias** | **Serious** |

## Dubreucq et al. (2020)

| **ROBINS-I domain** | **Judgment** |
| --- | --- |
| 1. Bias due to confounding | **Serious** |
| 2. Bias in selection of participants into the study | **Low** |
| 3. Bias in classification of interventions | **Low** |
| 4. Bias due to deviations from intended interventions | **Low** |
| 5. Bias due to missing data | **Moderate** |
| 6. Bias in measurement of outcomes | **Moderate** |
| 7. Bias in selection of the reported result | **Serious** |
| **Overall risk of bias** | **Serious** |

## Malchow et al. (2015)

| **ROBINS-I domain** | **Judgment** |
| --- | --- |
| 1. Bias due to confounding | **Serious** |
| 2. Bias in selection of participants into the study | **Low** |
| 3. Bias in classification of interventions | **Low** |
| 4. Bias due to deviations from intended interventions | **Low** |
| 5. Bias due to missing data | **Serious** |
| 6. Bias in measurement of outcomes | **Moderate** |
| 7. Bias in selection of the reported result | **Serious** |
| **Overall risk of bias** | **Serious** |

## Takahashi et al. (2020)

| **ROBINS-I domain** | **Judgment** |
| --- | --- |
| 1. Bias due to confounding | **Serious** |
| 2. Bias in selection of participants into the study | **Low** |
| 3. Bias in classification of interventions | **Low** |
| 4. Bias due to deviations from intended interventions | **Low** |
| 5. Bias due to missing data | **Serious** |
| 6. Bias in measurement of outcomes | **Low** |
| 7. Bias in selection of the reported result | **Moderate** |
| **Overall risk of bias** | **Serious** |

# RoB-2

## Choi et al. (2020)

| **RoB 2 domain** | **Judgment** |
| --- | --- |
| 1. Bias arising from the randomization process | **Some concerns** |
| 2. Bias due to deviations from intended interventions | **Low risk** |
| 3. Bias due to missing outcome data | **Low risk** |
| 4. Bias in measurement of the outcome | **Some concerns** |
| 5. Bias in selection of the reported result | **Some concerns** |
| **Overall risk of bias** | **Some concerns** |

## Dai et al. (2022)

| **RoB 2 domain** | **Judgment** |
| --- | --- |
| 1. Bias arising from the randomization process | **Some concerns** |
| 2. Bias due to deviations from intended interventions | **Low risk** |
| 3. Bias due to missing outcome data | **High risk** |
| 4. Bias in measurement of the outcome | **Low risk** |
| 5. Bias in selection of the reported result | **Some concerns** |
| **Overall risk of bias** | **High risk** |

## McGurk et al. (2021)

| **RoB 2 domain** | **Judgment** |
| --- | --- |
| 1. Bias arising from the randomization process | **Some concerns** |
| 2. Bias due to deviations from intended interventions | **Low risk** |
| 3. Bias due to missing outcome data | **Some concerns** |
| 4. Bias in measurement of the outcome | **Low risk** |
| 5. Bias in selection of the reported result | **Some concerns** |
| **Overall risk of bias** | **Some concerns** |

## Nuechterlein et al. (2023)

| **RoB 2 domain** | **Judgment** |
| --- | --- |
| 1. Bias arising from the randomization process | **Some concerns** |
| 2. Bias due to deviations from intended interventions | **Low risk** |
| 3. Bias due to missing outcome data | **Some concerns** |
| 4. Bias in measurement of the outcome | **Low risk** |
| 5. Bias in selection of the reported result | **Some concerns** |
| **Overall risk of bias** | **Some concerns** |

## Oertel-Knöchel et al. (2014)

| **RoB 2 domain** | **Judgment** |
| --- | --- |
| 1. Bias arising from the randomization process | **Some concerns** |
| 2. Bias due to deviations from intended interventions | **Low risk** |
| 3. Bias due to missing outcome data | **High risk** |
| 4. Bias in measurement of the outcome | **Low risk** |
| 5. Bias in selection of the reported result | **Some concerns** |
| **Overall risk of bias** | **High risk** |

## Shimada et al. (2025)

| **RoB 2 domain** | **Judgment** |
| --- | --- |
| 1. Bias arising from the randomization process | **Low risk** |
| 2. Bias due to deviations from intended interventions | **Low risk** |
| 3. Bias due to missing outcome data | **Low risk** |
| 4. Bias in measurement of the outcome | **Low risk** |
| 5. Bias in selection of the reported result | **Some concerns** |
| **Overall risk of bias** | **Some concerns** |

## Agostoni et al. (2025)

| **RoB 2 domain** | **Judgment** |
| --- | --- |
| 1. Bias arising from the randomization process | **Some concerns** |
| 2. Bias due to deviations from intended interventions | **Low risk** |
| 3. Bias due to missing outcome data | **High risk** |
| 4. Bias in measurement of the outcome | **Low risk** |
| 5. Bias in selection of the reported result | **Some concerns** |
| **Overall risk of bias** | **High risk** |
